# Decriminalization of cannabis use in South Africa: The perspectives and health outcomes among medical students; A systematic qualitative review

**DOI:** 10.1177/22799036251373016

**Published:** 2025-11-01

**Authors:** L. Winter Mokhwelepa, Gsakani Olivia Sumbane

**Affiliations:** 1 School of Medicine, Faculty of Health Science, University of Limpopo, Sovenga, Polokwane, South Africa

**Keywords:** cannabis, marijuana, decriminalization, medical students, health impacts, perspectives

## Abstract

**Background:**

The decriminalization of cannabis in South Africa has sparked growing concern within the medical community, particularly among medical students. As future healthcare providers, they must navigate evolving legal and professional landscapes while forming beliefs about the health effects of cannabis use. This legal shift has intensified debates surrounding the benefits, risks, and health consequences of cannabis, especially in relation to mental well-being, academic performance, and professional identity.

**Objective:**

This systematic qualitative review aimed to synthesize existing literature on South African medical students’ perceptions of cannabis decriminalization and their views on its health-related impacts. The review seeks to clarify how medical students interpret the effects of cannabis use on mental and physical health and how these beliefs shape their professional attitudes and behaviors.

**Design and methods:**

A systematic search and thematic synthesis were conducted across databases including PubMed, Scopus, PsycINFO, and ScienceDirect for literature published between 2010 and 2024. Studies were eligible if they focused on cannabis or marijuana use, included South African medical students, and discussed perspectives or health impacts in the context of decriminalization. Data were extracted, coded line-by-line, and synthesized to generate descriptive and analytical themes.

**Results:**

Four studies met inclusion criteria. Thematic analysis identified four key themes: (1) Health impacts (Mental and Physical); (2) perceptions and attitudes toward decriminalization; (3) educational influences and awareness; and (4) access to support services.

**Conclusion:**

This study emphasized the need for more focused research on the impact of cannabis decriminalization on medical students in South Africa. Current literature suggests that while there are diverse opinions on the subject, the decriminalization of cannabis may influence both attitudes and behaviors.

## Introduction

Cannabis use and its legal status remain highly contested across the globe, with countries navigating a complex interplay between public health priorities, legal reform, and social attitudes.^1^ In South Africa, cannabis was criminalized for decades, but a landmark Constitutional Court ruling in 2018 decriminalized its private use by adults.^2^ This shift has reignited debates over the health, legal, and social implications of cannabis, particularly in a country grappling with high rates of substance abuse, limited mental health services, and deep structural inequalities.^3^

While the decriminalization of cannabis has been hailed by some as a progressive step toward reducing criminal penalties and addressing social injustices, it has also raised critical public health questions.^4^ These include concerns about potential increases in cannabis use, normalization of recreational consumption, and insufficient regulation regarding its medical use.^5^ International evidence has suggested that decriminalization may correlate with increased use, particularly among youth and university students, a group that is already vulnerable to substance misuse due to academic pressures and psychosocial factors.^3,4^ In particular, cannabis use among university students has emerged as a significant concern due to its potential impact on cognitive functioning, academic performance, and mental health outcomes.^5,6^ Studies have linked cannabis consumption to reduced attention span, impaired memory, and lower academic achievement.^7,8^ Yet, despite this risk, young adults continue to be among the highest consumers of cannabis globally.^7^ Medical students occupy a unique position within this demographic. Not only are they exposed to the same stressors and social environments that may encourage substance use, but they also hold a future professional responsibility to prescribe, advise, and educate patients regarding drug use, including cannabis.^8^ As such, their attitudes, knowledge, and perceptions toward cannabis use particularly in the context of decriminalization deserve critical attention.

International literature sheds some light on this issue. In the United States and Serbia, studies by Chan et al. and Vujicic et al. found that a majority of medical students supported the legalization of cannabis and its use for medical purposes.^7,8^ These studies also highlighted that student who had previously used cannabis held more liberal views and demonstrated greater confidence in identifying its clinical applications. However, formal university education on cannabis pharmacology and regulation was notably lacking.^7,8^ Similarly, in Nigeria, a study of fifth-year medical students found that most held conservative attitudes, opposing the legalization of cannabis and perceiving it primarily as a harmful substance.^9^ These contrasting findings suggest that medical students’ perspectives are shaped by national contexts, including legal frameworks, cultural norms, and curriculum content.

Focusing exclusively on medical students is therefore justified, given their dual role as both current learners and future prescribers. Compared to other health professional students such as those in nursing or pharmacy, medical students receive more intensive pharmacological and clinical training and are often the primary point of contact for patient prescriptions and diagnoses.^10^ Their perceptions of cannabis use, and its health outcomes are likely to directly shape future prescribing patterns, public health messaging, and patient safety. While practicing medical professionals may offer more practice-based insights, medical students represent the generation that will inherit and navigate a shifting clinical landscape often without adequate educational resources or professional guidance.^11^ Furthermore, South Africa offers a particularly compelling case study due to its unique combination of socio-legal transitions, public health challenges, and educational gaps.^12^ High levels of youth unemployment, limited access to mental health care, and enduring inequalities intersect with the decriminalization of cannabis in ways that are not paralleled in many high-income countries.^1,2,12^ Understanding medical students’ views within this complex environment can provide critical insights into the preparedness of the future health workforce to address cannabis-related health needs and policy implications.

Despite the significance of cannabis decriminalization and its potential impacts on medical students, there remains a limited number of studies within the South African context. This gap in the literature makes it challenging to fully understand the perspectives and health outcomes experienced by medical students in relation to cannabis use. Therefore, this systematic review seeks to bridge this gap by synthesizing existing research to explore the perspectives of South African medical students on cannabis decriminalization and its health impacts, with the aim of identifying key trends and areas for further study. Moreover, the purpose of this study was to explore the impact of cannabis use on the health outcomes of medical students in South Africa, as well as to examine their perspectives on cannabis use following its decriminalization.

## Methodology

A systematic qualitative review was conducted to synthesize and critically evaluate existing research on the decriminalization of cannabis use in South Africa, with a specific focus on the perspectives and health outcomes among medical students. This methodology adhered to a structured approach, ensuring a rigorous and transparent synthesis of the available literature. A systematic qualitative review was chosen as it provides a comprehensive and structured approach to synthesizing existing literature, reducing bias, and ensuring a high level of rigor in the evaluation of research findings.^13,14^ This method allows for the identification of consistent patterns, gaps in knowledge, and emerging themes related to cannabis decriminalization and its impact on medical students. By systematically reviewing the literature, this study ensures that conclusions are based on a thorough and balanced assessment of the available evidence, thereby enhancing the reliability and applicability of the findings.

The Preferred Reporting Items for Systematic Reviews and Meta-Analyses (PRISMA) standard was followed in the conduct and reporting of this study.^14^ Although PROSPERO and the Cochrane Library were searched for comparable studies, none were located, despite the fact that this study was not registered with PROSPERO.

### Research question

What are the perspectives of medical students in South Africa regarding cannabis use following its decriminalization, and how does cannabis use affect their health outcomes?

### Search strategy

The literature search was conducted by the authors in December 2024. This systematic qualitative review included studies published in English between 2010 and 2024. The time frame was selected to reflect the period around and following the decriminalization of cannabis in South Africa, ensuring the inclusion of the most recent and contextually relevant literature on cannabis use and perceptions among medical students. A comprehensive search was conducted across four electronic databases: PubMed, Scopus, ScienceDirect, and PsycINFO. The search strategy combined controlled vocabulary (MeSH terms where applicable) and free-text keywords using Boolean operators (AND/OR) to maximize sensitivity and coverage across sources. Searches focused on three main domains: cannabis decriminalization, the target population (medical students), and their health-related outcomes.

Duplicate entries were removed using reference management software (e.g. EndNote), and additional relevant studies were identified through manual screening of the reference lists from eligible articles. Tables 1 and 2 demonstrate a full search strategy of this study.

**Table 1.** Search terms used.

Concept	Keywords and search terms
• Cannabis legal status	“Cannabis decriminalization” OR “marijuana decriminalization” OR “cannabis legalization” OR “marijuana legalization”
• Geographic scope	“South Africa”
• Target population	“Medical students” OR “undergraduate medical students”
• Health-related outcomes	“Health outcomes” OR “mental health” OR “cognitive effects” OR “psychological effects” OR “well-being”

**Table 2.** Databases searched and number of records retrieved.

Database	Number of records retrieved	Notes (e.g. filters, duplicates removed)
PubMed	124	Filters: English, 2010–2024
Scopus	147	Filters: English, 2010–2024; peer-reviewed journals only
ScienceDirect	98	Filters: English, 2010–2024; health sciences focus
PsycINFO	76	Filters: English, 2010–2024; psychology and medical education
Total	445	Before duplicates removed

**Figure 1.**
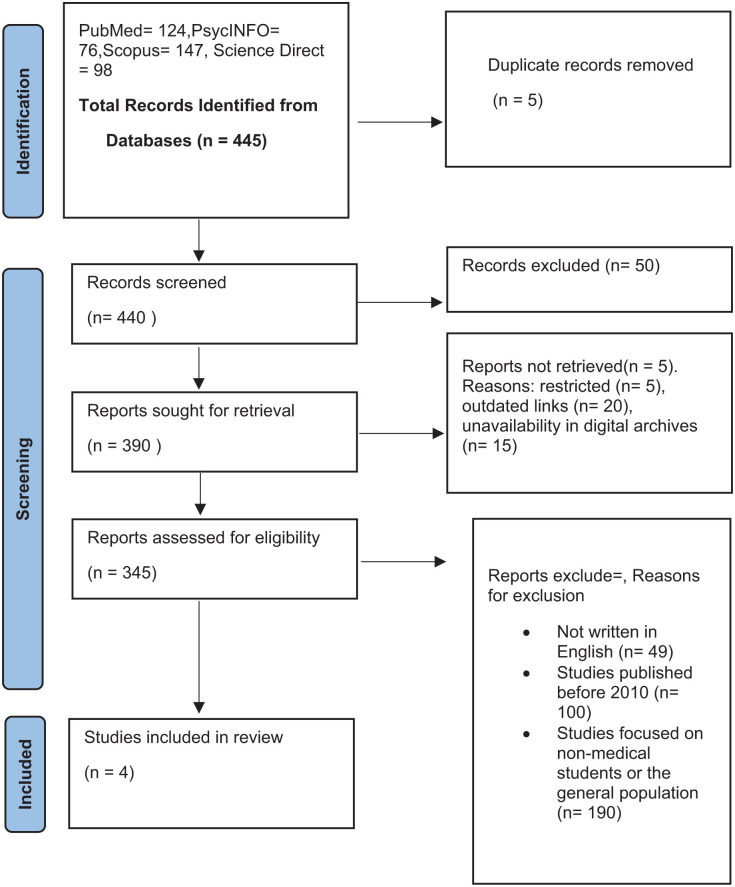
PRISMA flow diagram.^14^

To enhance transparency and reproducibility, a detailed PRISMA flow diagram (Figure 1) is provided, illustrating each stage of the study selection process. This includes the total number of records identified through database searching, duplicates removed, records screened, full-text articles assessed for eligibility, and studies included in the final qualitative synthesis. Reasons for exclusion at the full-text review stage are also specified. A total of 445 records were identified through systematic searches in four electronic databases: PubMed (124), PsycINFO (76), Scopus (147), and ScienceDirect (98). After removing five duplicate records, 440 unique records were screened based on titles and abstracts. During this initial screening, 50 records were excluded for not meeting the inclusion criteria.

Following screening, 390 full-text reports were sought for retrieval. However, five reports were not retrieved due to restricted access (5), outdated links (20), and unavailability in digital archives (15). These reasons resulted in a final 345 full-text reports being assessed for eligibility. Of these, 339 studies were excluded for the following reasons: not written in English (49), published before 2010 (100), and focused on populations other than medical students or the general population (190). Ultimately, four studies met all eligibility criteria and were included in this systematic qualitative review.

### Inclusion and exclusion criteria

Studies were included in this review if they were published between 2010 and 2024 and focused specifically on cannabis use among medical students in South Africa. Eligible studies examined the health, social, or psychological impacts of cannabis use among medical students in South Africa, as well as those that addressed decriminalization or relevant policy changes within the South African context. Studies were excluded if they focused on populations other than medical students, such as the general public or students from non-medical disciplines. Additionally, articles that centered solely on the medicinal use of cannabis, rather than recreational or personal use, were not considered. Only studies published in English were included in order to ensure consistent analysis and interpretation.

Medical professionals were excluded to maintain a focused scope on students in training, whose perceptions and behaviors may significantly differ from those of medical students. As future prescribers and public health advocates, medical students occupy a unique transitional space, and understanding their perspectives offers insight into the preparedness of the next generation of healthcare providers in the context of evolving cannabis policy in South Africa.

### Quality assessment

In this systematic review, the Newcastle-Ottawa Scale (NOS) was used to assess the quality of observational studies included in the analysis. This scale evaluates the quality of studies across five categories: sampling strategy, data collection, outcome measurement, statistical analysis, and bias control.^15^ Each category is scored from 0 (low), 1 (moderate) to 2 (higher), with higher scores reflecting better quality across all categories. Upon re-evaluation, the included studies varied in quality, with most receiving scores ranging from moderate to high. While three studies achieved scores close to the maximum, one study were rated as moderate due to limitations in sampling methods or potential bias in data collection. This updated assessment reflects a more nuanced evaluation, avoiding the overstatement that all studies achieved perfect scores. The quality appraisal table provided indicates these distinctions, ensuring that only studies with acceptable methodological standards were included in the synthesis. See Table 3 below.

**Table 3.** Quality appraisal tool.

Study	Study design/method	Sampling strategy (0–2)	Data collection (0–2)	Outcome measurement (0–2)	Statistical analysis (0–2)	Bias control (0–2)	Total score (0–10)	Quality rating
Vorster et al.^18^	Quantitative	2	2	2	2	2	10/10	High
van Zyl et al.^19^	Quantitative	2	2	2	2	2	10/10	High
du Plessis^20^	Quantitative	2	2	2	2	2	10/10	High
Levin et al.^21^	Quantitative		2	2	1	2	9/10	Moderate

To ensure the reliability of the quality assessment, two independent reviewers conducted the evaluation of all included studies. Each reviewer scored the studies separately based on the NOS criteria. Following the independent assessments, the reviewers compared their scores and discussed any discrepancies to reach a consensus. This collaborative process helped to minimize individual bias, clarify any ambiguities in the assessment criteria, and ensure consistent application of the quality standards across studies. By resolving differences through discussion, we enhanced the validity and credibility of the quality appraisal, thereby strengthening the methodological rigor of this review.

### Risk of bias

The risk of bias refers to the potential for systematic errors or distortions in the results of a study that may arise from various factors such as how the study was designed, how data were collected, or how the analysis was conducted.^16^ In this context, the bias control category specifically addresses how well the study controlled for potential biases, including selection bias, measurement bias, and confounding variables. A score of 2 in this category means the study took adequate measures to minimize bias.

### Data extraction and analysis synthesis

The qualitative synthesis followed Cresswell’s approach to data analysis, specifically utilizing Tesch’s open coding method.^17^ This involved a systematic process of reading through all the extracted data to gain a general sense, followed by detailed coding of each segment to identify key concepts and patterns. Two reviewers independently conducted open coding to ensure reliability. Codes were then organized into categories through a process of constant comparison. A consensus meeting was held to discuss discrepancies, refine categories, and agree upon the final themes. This iterative process allowed for the emergence of core themes that reflect the perspectives and health outcomes related to cannabis use and its decriminalization among South African medical students. The use of Tesch’s method ensured a rigorous and grounded thematic structure.^17^ Therefore, four main themes emerged in this study. Table 4 below shows summary characteristics and data extraction.

**Table 4.** Summary characteristics and data extraction.

Author(s) and study title	Study aim/objective	Study method	Population (*n*)	Sex of the participants (M/F)	Country	Thematic area	Key findings related to review aim/objective
Vorster et al., 2019^18^ *Second-and third-year medical students’ self-reported alcohol and substance use, smoking habits and academic performance at a South African medical school.*	This study aimed to determine self-reported use of alcohol, illicit substances (e.g. cannabis, lysergic acid diethylamide (LSD), magic mushroom, cocaine, crack, ecstasy, methamphetamine and heroin), prescription medication and smoking habits, correlating academic performance.	Quantitative (observational, descriptive, cross-sectional design)	171	Both	South Africa	Substance Use and Academic Impact	High prevalence of cannabis use
van Zyl et al., 2017.^19^ *Depression, anxiety, stress and substance use in medical students in a 5-year curriculum.*	To determine the prevalence of depression, anxiety, stress and substance use among preclinical students in a 5-year outcomes-based medical curriculum.	Quantitative (cross-sectional design)	295	Both	South Africa	Mental Health and Coping	Increased levels of stress
du Plessis.^20^ *A survey of the knowledge and perceptions of South African medical practitioners concerning the use of medical cannabis by patients.*	To identify the knowledge and perceptions concerning medical cannabis for palliative care patients amongst South African doctors.	Quantitative	50	Both	South Africa	Clinical Knowledge and Perceptions	More information was obtained on the importances of cannabis use among patients
Levin et al., 2010^21^ *Dagga (cannabis) usage among medical students in Johannesburg.*	To determine the effects of cannabis usage among medical students in Johannesburg.	Quantitative (cross-sectional study)	1020	Both	South Africa	Coping Mechanisms and Health Outcomes	Cannabis was used as a coping mechanism

To enhance methodological rigor, the coding process included measures to ensure coder reliability. Both reviewers independently coded the data and then compared their coding to identify any inconsistencies. Discrepancies were discussed in detail during consensus meetings until full agreement was reached on code definitions and theme categorizations. This approach minimized subjective bias and increased the trustworthiness of the thematic analysis by ensuring that the themes accurately represented the data.

## Results

Through thematic synthesis of the four included studies, four core themes emerged that reflect the perceptions and experiences of South African medical students in relation to cannabis decriminalization and its health-related implications. These themes are: (1) Health Impacts (Mental and Physical); (2) Perceptions and Attitudes Toward Decriminalization; (3) Educational Influences and Awareness; and (4) Access to Support Services. While the included studies used both quantitative methods, this review integrates their findings thematically to provide a holistic understanding of how decriminalization influences student behavior, attitudes, and perceived health consequences.

### Health impacts on medical students: Mental health and physical health

The literature reported that medical students experience both mental and physical health outcomes related to cannabis use. Mental health effects included increased reports of stress, anxiety, depression, and cognitive impairments among users.^17^ Cannabis use was also associated with decreased motivation and executive functioning, with some studies noting a link to psychotic symptoms in individuals with a predisposition.^17^ On the physical health front, frequent use particularly through smoking was associated with respiratory problems, increased heart rate, and potential cardiovascular risks.^17^ Altered appetite, disrupted sleep patterns, and weight fluctuations were also identified.

### Perceptions and attitudes: Views on decriminalization and impact on professionalism

The review found diverse attitudes among students regarding cannabis decriminalization. Some students viewed the policy shift as a step toward social progress, aligning it with individual freedom and public health approaches like harm reduction.^18,19^ For this group, cannabis was perceived as a low-risk substance, comparable to alcohol. However, other students expressed apprehension, citing risks of increased dependency, academic impairment, and ethical dilemmas.^19^ Concerns were also raised about cannabis use affecting clinical competence and professional judgment.^18^

### Educational impact: Curriculum influences, and knowledge and awareness

Findings indicate that the current medical curriculum inadequately addresses cannabis use and its health impacts. Some students reported limited training on the clinical implications of cannabis use, contributing to gaps in understanding its effects and appropriate professional conduct.^20^ There was inconsistency in the level of knowledge across institutions, with some students calling for curriculum reform to include content on substance use, mental health, and professional responsibility.^20,21^

### Access to support services

A recurring finding across studies was the limited access to adequate support services for medical students using cannabis. While some universities provided health and counseling services, barriers such as stigma, fear of professional consequences, and low awareness discouraged students from seeking help.^21^ The data suggested that institutional efforts to address substance use among students remain insufficient and require more proactive, stigma-free support mechanisms.^21^

## Discussion

This systematic qualitative review examined the perspectives of South African medical students on cannabis decriminalization and explored the health impacts associated with its use among medical students. The review found that most medical students held permissive attitudes toward cannabis legalization, particularly among male students and those with prior use. These students tended to have greater confidence in cannabis’s medical applications and were more likely to support broader legal reforms.^22,23^ Conversely, medical students who had never used cannabis expressed greater awareness of its potential side effects and risks, suggesting a divergence in knowledge depending on personal experience.^24,25^

These findings resonate with international research conducted in the United States and Serbia,^7,8^ where prior cannabis use similarly predicted more liberal views and higher knowledge about clinical applications. However, the South African context presents a unique intersection of legal reform, limited education, and high usage prevalence.^1^ The 2018 Constitutional Court ruling permitting adult personal use in private has likely contributed to changing student perceptions, creating a more permissive environment that contrasts sharply with more conservative views reported among medical students in Nigeria and other African countries.^9^ A study at the University of the Free State, for example, found that over 30% of medical students had used cannabis,^26^ indicating a higher prevalence than most comparable African contexts. This divergence may point to broader sociopolitical openness and differing enforcement mechanisms in South Africa.

A key finding of this review is the normalization of cannabis use as a stress-relief mechanism among students. While many students cite academic pressure, burnout, or insomnia as motivations for cannabis use,^27–29^ this normalization is worrying. The literature consistently links regular cannabis use to impaired executive functioning, reduced concentration, memory problems, and poorer academic performance.^23,30–32^ These consequences are particularly detrimental for medical students, for whom sustained cognitive performance and ethical professionalism are vital.

South African studies echo global evidence that prolonged cannabis use can contribute to mental health disorders, including increased rates of anxiety, depression, and in some cases, psychosis especially among those with a family history of such conditions.^33,34^ The review highlights that some students perceive cannabis as less harmful than alcohol or tobacco, revealing a potential underestimation of its risks, especially regarding long-term mental health and neurocognitive development.^34^

The influence of media and informal sources, rather than structured university education, was another notable trend identified in the review. In the Serbian study by Vujicic et al.,^8^ and echoed in South African contexts, students primarily learned about cannabis from the internet and social media. This lack of formal education on cannabis whether clinical, legal, or ethical raises concerns about whether future doctors are adequately equipped to counsel patients, navigate legislation, or manage their own substance use in a professional context.^35^

In terms of professional development, cannabis use among medical students raises questions about the broader implications for clinical judgment and ethical behavior. The review noted that increased cannabis use may blur boundaries between personal freedom and professional responsibility.^36,37^ Regular use could undermine public trust and may have implications for patient safety, particularly if students enter clinical practice with unresolved dependencies or misinformation about the substance.^38^

This review also exposes a critical gap in South African literature, very few studies focus on cannabis use within this specific academic population. While broader population surveys exist, the perspectives and behaviors of future health professionals have not been thoroughly explored. Furthermore, the review identified only a small number of studies that assessed the impact of decriminalization on behavior change among students, suggesting a need for longitudinal studies that track changes in use patterns, attitudes, and academic outcomes.

### Implications for policy and education

Given the findings, there is a clear need for medical curricula to integrate comprehensive, evidence-based substance use education, not only from a clinical standpoint but also to support students’ own well-being and professional growth. Universities should consider implementing tailored health promotion programs, as well as counseling and support services that address substance use, mental health, and stress management. Policymakers, too, must recognize that decriminalization while progressive requires parallel investments in public education and harm reduction strategies, particularly within high-risk groups like students.

### Study limitations

This systematic qualitative review on the decriminalization of cannabis use in South Africa and its impact on medical students is subject to several limitations. Firstly, the geographic and population focus narrowed the pool of eligible studies, resulting in a limited number of articles specifically addressing South African medical students, which may affect the generalizability of the findings. Secondly, due to the qualitative nature of the review, there is potential for interpretative bias during thematic analysis, as the synthesis depends heavily on the researchers’ subjective interpretation of the data. The inclusion of only English-language publications may have excluded important perspectives documented in other languages. Furthermore, given the evolving legal and social landscape surrounding cannabis use in South Africa, some findings may quickly become outdated, highlighting the need for continuous updates as new research emerges. Lastly, a key limitation of this study is the absence of a formal sample size calculation, which is typically more applicable in primary quantitative research. In qualitative systematic reviews, the emphasis is placed on the depth and richness of data rather than statistical power. Nevertheless, this limitation should be acknowledged as it may influence the generalizability and comprehensiveness of the findings.

## Conclusion

In conclusion, this systematic review revealed the significant mental, physical, and academic impacts of cannabis use among South African medical students, especially in the context of its decriminalization. The limited research on this topic underscores the need for further studies to explore the long-term consequences of cannabis use in medical education. Addressing the stigma surrounding substance use, enhancing support services, and incorporating health education into curricula are critical steps in mitigating the risks and promoting healthier behaviors among medical students.

## Supplemental Material

sj-docx-1-phj-10.1177_22799036251373016 – Supplemental material for Decriminalization of cannabis use in South Africa: The perspectives and health outcomes among medical students; A systematic qualitative review Supplemental material, sj-docx-1-phj-10.1177_22799036251373016 for Decriminalization of cannabis use in South Africa: The perspectives and health outcomes among medical students; A systematic qualitative review by L. Winter Mokhwelepa and Gsakani Olivia Sumbane in Journal of Public Health Research
